# Great minds think different: Preserving cognitive diversity in an age of gene editing

**DOI:** 10.1111/bioe.12585

**Published:** 2019-04-02

**Authors:** Jonathan Anomaly, Christopher Gyngell, Julian Savulescu

**Affiliations:** ^1^ University of California at San Diego San Diego CA USA; ^2^ University of Melbourne and Melbourne Law School Victoria Australia; ^3^ Oxford Uehiro Centre for Practical Ethics University of Oxford UK

**Keywords:** cognitive diversity, embryo selection, gene editing, genetic enhancement, regulatory parsimony

## Abstract

It is likely that gene editing technologies will become viable in the current century. As scientists uncover the genetic contribution to personality traits and cognitive styles, parents will face hard choices. Some of these choices will involve trade‐offs from the standpoint of the individual's welfare, while others will involve trade‐offs between what is best for each and what is good for all. Although we think we should generally defer to the informed choices of parents about what kinds of children to create, we argue that decisions to manipulate polygenic psychological traits will be much more ethically complicated than choosing Mendelian traits like blood type. We end by defending the principle of regulatory parsimony, which holds that when legislation is necessary to prevent serious harms, we should aim for simple rules that apply to all, rather than micro‐managing parental choices that shape the traits of their children. While we focus on embryo selection and gene editing, our arguments apply to all powerful technologies which influence the development of children.

## 1 INTRODUCTION

A commonly expressed fear in dystopian novels and popular debates about genetic engineering is that we will produce future people who are exactly alike. But part of the beauty of life is the astonishing diversity that evolution has produced. Of course, evolution has also left us with genetic disorders like Huntington's disease that we'd be better off without. But the difference between eliminating simple genetic disorders and selecting non‐disease genes that influence psychological styles is big. For one thing, important aspects of our psychology ranging from general intelligence to specific personality traits like extraversion are polygenic—they result from many genes with small effects interacting with one another.1
Plomin, R., & Deary, I. J. (2015). Genetics and intelligence differences: Five special findings. *Molecular Psychiatry,* 20, 98–108.10.1038/mp.2014.105PMC427073925224258
 For another, many traits that seem undesirable in some ways are desirable in others. Introversion, for example, may lead to less subjective well‐being than extraversion but also to artistic creation.2
Nettle, D. (2006). The evolution of personality variation in humans and other animals. *American Psychologist*, 61, 622–631.10.1037/0003-066X.61.6.62216953749
 To the extent that some traits might reduce subjective well‐being but have compensating individual or social benefits, there are difficult ethical decisions about whether to preserve, reduce, or enhance them.

Science fiction raises a legitimate question: how do we preserve a diversity of people in a world in which parents can influence the genetic contributions to their children's personality? This question motivates the article. In Part 2 we review recent research that examines the genetic basis of psychological traits, focusing on personality traits and political orientation. In Part 3 we review evidence for the benefits of cognitive diversity for groups, and in Part 4 show how the development of enhancement technologies will lead to trade‐offs for each and moral dilemmas for all. In Part 5 we consider and reject the claim that the existence of such trade‐offs generates reasons to abstain from intervening in ways that shape personality traits. In Part 6 we introduce our favored strategy—regulatory parsimony.

## 2 HERITABILITY OF PSYCHOLOGICAL TRAITS

Scientists do not yet understand the precise role that genes play in the development of most psychological traits,3
We use “psychological traits” as a general term that includes specific cognitive abilities, personality traits, and affective dispositions.
 so our discussion is speculative. But as the use of technology to select and alter embryos is likely to become ubiquitous, it is worth raising these issues before the science is complete. Behavioral genetics is the systematic study of how genes contribute to behavior, including behavior associated with personality type. It uses the comparison of monozygotic and dizygotic twins to infer the heritability of traits.4
The best general discussion of heritability is Sesardic, N. (2005). *Making sense of heritability*. Cambridge: Cambridge University Press.
 Behavioral genetics is an indirect way of understanding the genetic underpinnings of behavior in the absence of detailed knowledge of how genes shape behavior. As computational biology improves, we will learn more about the relationships between specific genetic variants and the kinds of behavior they predispose us toward.

Although we will focus on the heritability of specific psychological traits we should first emphasize what is arguably the most important determinant of health, wealth, and welfare: general intelligence.5
Sandberg, A., & Savulescu, J. (2011). The social and economic impacts of cognitive enhancement. In J. Savulescu, R. Ter Meulen, & G. Kahane, (Eds.), *Enhancing human capacities* (pp. 92–112). Oxford: Wiley‐Blackwell.
 According to a widely shared view in quantitative psychology, “Intelligence is one of the best predictors of key outcomes such as education and occupational status. People with higher intelligence tend to have better mental and physical health and fewer illnesses throughout the life course, and longer lives.”6
Plomin & Deary, op cit. note 1, p. 99.
 Intelligence is also correlated with a range of other qualities, including patience and conscientiousness, which have a substantial impact on a host of life outcomes. Higher intelligence is negatively correlated with crime7
Beaver, K., Schwartz, J. A., Nedelec, J. L., Connolly, E. J., Boutwell, B. B., & Barnese, J. C. (2013). Intelligence is associated with criminal justice processing. *Intelligence*,* 41*(5), 277–288.
 and positively correlated with cooperation in strategic interactions that require patience and long‐term planning.8
Jones, G (2008). Are smarter groups more cooperative? *Journal of Economic Behavior and Organization*,* 68*(3–4), 489–497.
 It appears that intelligence is an all‐purpose good that many parents will want to enhance in their children, at least up to a point.9
Apart from the private reasons parents have, if general intelligence has positive network effects, predicting social trust and cooperation, there may be good moral reasons to enhance intelligence (Anomaly & Jones, Cognitive enhancement and network effects; in preparation).
 But we want to focus on a neglected topic in the biomedical enhancement debate: specific psychological traits that are heritable and that may involve trade‐offs with other psychological traits.

### 2.1 Personality traits

There are a variety of personality types in the human population, and nobody pretends we can neatly divide everyone into a few types. But the Big Five personality inventory is a widely used way to think about personality variation and the heritability of personality traits. The Big Five are openness, conscientiousness, extraversion, agreeableness, and neuroticism.

On most accounts, each of the Big Five has a substantial genetic component, with genes accounting for an average of about 40–60% of the variation between individuals in a population.10
Bouchard, T. (2004). Genetic influence on human psychological traits. *Current Directions in Psychological Science, 13*(4): 148–151; Polderman, T., Benyamin, B., de Leeuw, C. A., Sullivan, P. F., van Bochoven, A., Visscher, P.… Posthuma, D. (2015). Meta‐analysis of the heritability of traits based on fifty years of twin studies. *Nature Genetics*,* 47*(), 702–712.10.1038/ng.328525985137
 The heritability of general intelligence is closer to 80% by adulthood.11
Haier, R. (2016). *The neuroscience of intelligence*. Cambridge: Cambridge University Press.
 One implication is that while environments matter, genetic influences on personality are powerful. Another is that intelligence and personality can be sculpted—if not fully determined—by mate selection, embryo selection, and (potentially) genetic engineering.

Extraversion generally has a positive connotation, while neuroticism has a negative connotation. But these are technical terms in psychology that reflect traits that can be described as good or bad for human welfare only in a broader context. So we should avoid thinking about the ordinary connotations of these terms, or their tendency to increase or reduce reproductive fitness in ancestral conditions, and instead focus on how they are likely to affect the welfare of people in the modern world. In the following few paragraphs we summarize some results from an overview of the Big Five by Daniel Nettle.12
Op cit. note 2.

- Openness is associated with seeking novelty and making associations between disparate domains. It is correlated positively with creativity but is also mildly associated with delusion, depression, and even schizophrenia;13
The association seems to be genetic: Close relatives of schizophrenics are more likely to be in creative professions. See Plomin, R. (2018). *Blueprint: How DNA makes us who we are*. New York: Penguin, p. 151.

- Conscientiousness is associated with the ability to delay gratification in the pursuit of goals. Conscientious people tend to be good at figuring out how to execute a plan, and they also tend to avoid antisocial behavior;
- Extraversion is associated with having an active social life, and seeking out new opportunities. It is also associated with having more sexual partners. These traits confer benefits, but they also tend to increase the risk of danger, and indeed, in the modern world extraversion is positively (though weakly) correlated with being involved in criminal or antisocial behavior;
- Agreeableness is associated with empathy and trust, and with harmonious personal relationships. People who score high on conscientiousness and agreeableness tend to be good friends, and good partners in coalitions, in part because they pay attention to the needs of others, and can be counted on to stick to the agreements they make. The main downsides to conscientiousness and agreeableness is that they can make an individual liable to exploitation by free riders who seek to manipulate their trust and empathy;
- Neuroticism is associated with negative emotional attitudes like fear, guilt, anxiety, and depression. But neuroticism interacts with other traits in positive and negative ways. For example, when neuroticism is paired with intelligence and conscientiousness, it can lead to achievement in professional and academic settings. When it is not, or when the environment stifles opportunities to exercise one's abilities, it can lead to crippling anxiety or depression.

### 2.2 Pathological personality traits

#### 2.2.1 Scrupulosity

Many personality traits are healthy in moderate amounts but pathological in large amounts. For example, to the extent that social norms help members of groups coordinate their behavior, it's good for all if each member understands, follows, and enforces social norms. Enforcing a norm may seem like a prisoner's dilemma, as each enforcer bears the cost of identifying and sanctioning those who violate rules, while the benefits of norm enforcement are dispersed among all members of a group. But natural selection seems to have partially solved the problem by equipping us with moral emotions that motivate us to follow norms and sanction norm violators. Guilt and shame make us inclined to punish ourselves for flouting socially beneficial rules and indignation leads us to want to punish others who break them.14
Bowles, S., & Gintis, H. (2011). *A cooperative species*. Princeton, NJ: Princeton University Press.



But there is natural variation in people's propensity to follow and enforce norms. Some people have a strong propensity to punish rule violators and others have very little inclination to do so. It is socially beneficial to have norm enforcers in a population to promote cooperation. But an extreme version of this personality trait, which psychopathologists call scrupulosity, occurs when some members of a group obsessively enforce rules, looking to punish every minor violation with tough sanctions. This can make the individual who has the quality frustrated, especially when she fails to be able to understand that the spirit of the law is more important than the letter of the law. And it can make the group worse off as it's inefficient to have people devoting every bit of energy obsessing over rules rather than living a productive and happy life.

The excessively scrupulous person—an extreme form of conscientiousness—cares too much about social conventions, treating them as intrinsically important and inflexible rather than a malleable way of promoting social welfare. On the other side, people who are inclined to flout conventions can benefit a population. As some norms are oppressive, having some non‐conformists in a population will tend to promote social progress even if it's good for most people to follow rules that may increase predictability and harmony. But having an obsessive propensity to follow rules or having no concern for rules at all can be individually crippling and socially harmful.

#### 2.2.2 Autism

The characteristics of some people on the autistic spectrum may be another example of traits that are beneficial in moderate degrees but harmful in extreme degrees. Some of the diagnostic criteria of autism include a tendency to systematize and classify, a severely impaired ability to understand social cues, and relatively low levels of empathy.15
Baron‐Cohen, S. (2006). Two new theories of autism: Hyper‐systematizing and assortative mating. *Archives of Disease in Childhood*,* 91*(1), 2–5.10.1136/adc.2005.075846PMC208310216371371
 Some researchers have pointed out that these characteristics can be viewed as an extreme version of the male brain, which tends to excel at systematizing more than empathizing.16
Ploeger, A., & Galis, F. (2011). Evolutionary approaches to autism—an overview and integration. *McGill Journal of Medicine*,* 13*(2), 38.PMC327741322363193
 Couples who score relatively high on systematizing are more likely to have children with autistic qualities than those who don't.17
Op cit. note 15.
 While severe forms of autism can lead to a life of dependence and frustration, some of the genes that contribute to autism may contribute to a personality profile of someone with especially high ability, particularly in engineering and mathematics.18
Baron‐Cohen, S. (2012). *Zero degrees of empathy: A new theory of human cruelty and kindness*. New York: Penguin, p. 98.
 The presence of these personality traits in our society may contribute to important social goods.19
Gyngell, C., & Douglas, T. (2018). Selecting against disability: The liberal eugenic challenge and the argument from cognitive diversity. *Journal of Applied Philosophy*,* 35*(2), 319–340.



Some psychologists have distinguished between cognitive empathy, which allows us to understand what other people are thinking, and affective empathy, which leads us to want to help other people who are in distress. People with low‐functioning autism often score low in both kinds of empathy. Others have challenged this view, arguing that most autistic people have affective empathy but test low on cognitive empathy.20
Mazza, M., Pino, M. C., Mariano, M., Tempesta, D., Ferrara, M., De Berardis, D., … Valenti, M. (2014). Affective and cognitive empathy in adolescents with autistic spectrum disorder. *Frontiers in Human Neuroscience*,* 7*(8), 791.10.3389/fnhum.2014.00791PMC418757925339889



#### 2.2.3 Psychopathy

In contrast, psychopaths excel in cognitive empathy, but lack a desire to help others unless it's to their own benefit. According to Simon Baron‐Cohen, “the psychopath is aware that they are hurting someone because the ‘cognitive’ (recognition) element of empathy is intact in their case, even if the ‘affective’ element (the emotional response to someone else's feelings) is not".21
Op cit. note 18, p. 85; Correction added on 25 April 2019, after first publication: The preceding quotation was previously wrong and has been corrected in this version.



Psychopaths are dangerous because they understand how to get what they want, and manipulate others in the process, but don't care about the pain they inflict on other people. Psychopathy is highly heritable and, like most character traits, polygenic.22
Tuvblad, C., Bezdjian, S., Raine, A., & Baker, L. A. (2014). The heritability of psychopathic personality in 14–15 year old twins: A multirater, multimeasure approach. *Psychological Assessment*,* 26*(3), 704–716.10.1037/a0036711PMC415540724796343
 At the opposite end of psychopathy, people with very high levels of affective empathy ‐‐strongly influenced by genes and hormones ‐‐are more susceptible to anorexia, bipolar disorder, and schizophrenia..23
Warrier, V., Toro R., Chakrabarti B., iPSYCH‐Broad autism group, Børglum A. D., Grove J., … Baron‐Cohen, S. (2018). Genome‐wide analyses of self‐reported empathy. *Translational Psychiatry*,* 8(1),* 35.10.1038/s41398-017-0082-6PMC584586029527006



### 2.3 Political orientation

It may seem odd to argue that political orientation is heritable. After all, plenty of people hold completely different political views from their parents or siblings. But it is important to understand that all personality traits are heritable to some degree, and some traits incline us more toward some political ideologies than others. Jonathan Haidt summarizes the process by saying: genes make brains, which come pre‐wired with personality traits; traits lead children to take different life paths, and these different paths lead us to construct life narratives that fit a non‐random subset of the available political ideologies.24
Haidt, J. (2012). *The righteous mind: Why good people are divided by politics and religion*. New York: Vintage, Chapter 12.
 This should not be surprising as politics is a human construct: our political ideologies reflect what we care about and how we tend to view the world. In fact, the heritability of political orientation tends to grow after young adults leave home because this is when they have the greatest capacity to seek out and mold environments that accord with their innate predispositions.25
Hatemi, P., & McDermott, R. (2012). The genetics of politics: Discovery, challenges, and progress. *Trends in Genetics*,* 28*(10), 525–533.10.1016/j.tig.2012.07.00422951140
 While parents can strongly influence their children's political orientation when they're young, these effects tend to dissipate as their children increasingly choose environments and ideologies that match their personality better.

In thinking about how genes influence political orientation:

[T]he most likely answer is not that attitudes on specific issues are heritable, but that issue positions reflect a set of heritable core predispositions, including values and personality traits. These core predispositions, which are influenced by life experiences as well as genes, are used by individuals to navigate the social, economic, and political worlds and as such serve as the basis for specific attitudes on issues of the day.26
Funk, C., Smith, K. B., Alford, J. R., Hibbing, M. V. Eaton, N. R., Krueger, R. F. … & Hibbing, J. R. (2013). Genetic and environmental transmission of political orientations. *Political Psychology*,* 34*(6), 805–819, p. 806.

Genes that influence political orientation are an example of genetic factors that affect phenotypes via “outside the skin” pathways.27
Kendler, K., & Ralph, G. (2006). The nature of genetic influences on behavior: Lessons from “simpler” organisms. *American Journal of Psychiatry*,* 163*(10), 1687.10.1176/ajp.2006.163.10.168317012675
 They influence the way we move through our social and cultural environments, which in turn change the specific values and ideologies we adopt.

To some extent, the Big Five correlate with political orientation. For example, a liberal political orientation strongly correlates with the personality trait of openness.28
Op cit. note 24.
 But the best evidence so far is that political orientation is a separate dimension of personality.29
Hatemi, P., Medland, S. E., Klemmensen, R., Oskarsson, S., Littvay, L., Dawes, C. T. … Martin, N. G. (2014). Genetic influences on political ideologies: Twin analyses of 19 measures of political ideologies from five democracies and genome‐wide findings from three populations. *Behavioral Genetics*,* 44*(3), 282–294.10.1007/s10519-014-9648-8PMC403893224569950
 Like other personality traits, “most researchers consider political traits to be influenced by thousands of genetic markers both indirectly and through interactions with numerous environmental stimuli and other genes.”30
Op cit. note 25, p. 527.



Behavioral geneticists have pointed out that assortative mating—the tendency to choose long‐term mates with similar psychological traits—is especially high for intelligence,31
Op cit. note 1.
 and may be growing in countries in which women are free to become professionals and where professionals are more likely to marry each other than to marry people with lower status jobs.

Perhaps more striking than assortative mating for intelligence, “long‐term mates correlate more highly on political ideologies (.65–.71) than on almost any other clinical, behavioral, or psychological trait.”32
Op cit. note 25, p. 527.
 Some worry that an increase in assortative mating for political orientation may exacerbate the political divide.33
Alford, J., Funk, C. L., & Hibbing, J. R. (2005). Are political orientations genetically transmitted? *American Political Science Review*,* 99*(2), 165.
 But the strong correlation for political orientation may not be as worrisome as it seems. It may be that the correlation between long‐term mates and political orientation is not explained simply by the fact that partners have similar personality traits. It may partly be explained by the fact that in many places political orientation is a kind of ethnic marker, like race and religion. In fact, there is evidence that in the USA today people are at least as likely to discriminate against others of a different political party than against others of a different race or religion.34
Iyengar, S., & Westwood, S. (2015). Fear and loathing across party lines: New evidence on group polarization. *American Journal of Political Science*,* 59*(3), 690–707.
 It is conceivable that quick and accurate gene sequencing combined with embryo selection or gene editing technology could increase the genetic basis of existing political divides.

We have tried to give an overview of how different psychological traits are both heritable and capable of producing different life outcomes and sociopolitical arrangements. We now want to explore why cognitive diversity is desirable, and what kinds of trade‐offs we might face in selecting or altering embryos to shape our children's traits.

## 3 THE VALUE OF COGNITIVE DIVERSITY

A number of authors have emphasized that if we want to solve a complex problem it is often better to have a cognitively diverse group of people than the same number of very bright people who see the world in a similar way.35
Hong, L., & Page, S. (2004). Groups of diverse problem solvers can outperform groups of high ability problem solvers. *Proceedings of the National Academy of Sciences of the United States of America*,* 101*(46), 16385–16389.10.1073/pnas.0403723101PMC52893915534225
 This insight is perhaps best captured by William Buckley's quip that he'd “rather entrust the government of the United States to the first 400 people listed in the Boston telephone directory than to the faculty of Harvard University.”36
Wakefield, D. (1961). William F. Buckley: Portrait of a complainer. New York: *Esquire*.
 People with different experiences and different cognitive styles (which arise from a confluence of psychological traits), look at the world through different lenses that can complement one another. And as long as they are organized in teams or in groups that communicate and cooperate effectively their different talents can be harnessed to solve complex problems.

Apart from simple teamwork, Jonathan Haidt has given some reason to believe that people with diverse cognitive styles may be better at solving political problems.37
Op cit. note 24.
 For example, people with conservative personalities tend to be skeptical of outsiders and of radical new ways of organizing political society and liberals tend to be more open to alternatives to the existing arrangements. A healthy proportion of liberals in a population can help a group learn from outsiders and allow people to try out new experiments in living (to paraphrase John Stuart Mill). But conservatives may have a beneficial influence in pressing the brakes when radical alternatives are likely to expose a group to danger.38
Conservatives also tend to emphasize values and family structures that have historically fostered above replacement‐level fertility, whereas liberal societies have seen their numbers plummeting to sub‐replacement levels. As Filipe Faria has argued, market liberalism may be creating a self‐defeating pattern of fertility declines in the most prosperous societies. This is not necessarily an argument for returning to a more conservative society, but it may be yet another interesting trade‐off between groups comprised of people with different values and cognitive traits. See Faria, F. (2017). Is market liberalism adaptive? *Journal of Bioeconomics, 19(*3), 307–326.



Similarly, cognitive diversity can combine with open societies to produce salutary social effects. Mill argued that even people who are content with the status quo often benefit from new ways of thinking and models of living:

Originality is the one thing which unoriginal minds cannot feel the use of [.… But] recollecting that nothing was ever yet done which someone was not the first to do, and that all good things which exist are the fruits of originality, let them be modest enough to believe that there is something still left for it to accomplish, and assure themselves that they are more in need of originality, the less they are conscious of the want.39
Mill, J. S. (1859). On Liberty: Reprinted in New York by Penguin, Chapter 3.

While Mill's point is about originality and eccentricity, these are at least partly shaped by different psychological traits as well as different environments. Diversity is good, on this view, to the extent that it can help us solve political problems and come up with cultural and scientific innovations that benefit everyone. More recently, Jerry Gaus has extended Mill's insight to argue that some degree of moral diversity, which is tied to cognitive diversity, can help populations solve social and political problems that arise in response to an increasingly complex world.40
Gaus, G. (2018). The complexity of a diverse moral order. *Georgetown Journal of Law and Public Policy*,* 16*(S).
 It is important, though, not to overplay this point. Radical diversity of values in a population is likely to undermine trust and impair successful collective action.41
Ostrom, E. (2000). Collective action and the evolution of social norms. *Journal of Economic Perspectives*,* 14*(3), 137–158.
 What is important is that there is some optimum range of diversity.42
Kahane, G., & Savulescu, J. (2014). Normal variation: Refocusing the enhancement debate. *Bioethics*,* 29*(2), 133–143.10.1111/bioe.12045PMC427883923906367



Finally, quite apart from their instrumental effects on solving political problems and generating new ideas in the arts and sciences, aesthetic and personality differences can make life more enjoyable (at least within certain parameters: having more psychopaths in the population more or people with severe autism is not especially desirable).

Given the value of diversity, and assuming there is some genetic contribution to it, we want to explore two kinds of trade‐offs. First, the trade‐offs that parents will face in choosing a child with different traits when they are concerned only with the child's welfare; and second, the trade‐offs parents will face between selecting or altering children in a way that is likely to affect the welfare of society more generally. The total effects of such trade‐offs may be small and difficult to predict for any given child. But from a social standpoint, the aggregate effects of many such choices are significant.

## 4 ENHANCEMENT DILEMMAS

Suppose we gain the power to alter embryos before they develop into children. Versions of gene editing technology such as CRISPR Cas9 already promise to make genetic engineering a reality in the near future, even if we're a long way off from manipulating polygenic psychological traits.43
Op cit. note 13, p. 116.
 Assuming this technology becomes widely available, we will face a number of hard choices. Even if gene editing does not progress, whole genome analysis of embryos is now possible and genetic selection for psychological traits is on the horizon. And there will be other technological and social means of modifying a child's personality (such as pharmaceuticals and medical devices). We will assume (rather than defend) two principles: procreative beneficence and procreative altruism. Although there are plenty of philosophical objections to these principles we will take them for granted in order to explore what they might imply about parental choices that may shape the characteristics of children.

According to the principle of procreative beneficence44
Savulescu, J. (2001). Procreative beneficence: Why we should select the best children. *Bioethics*,* 15*(5–6), 413–426.10.1111/1467-8519.0025112058767
 parents should select the child, of the children they could have, who is expected to live the best life possible (in terms of well‐being), given the available information. According to the principle of procreative altruism45
Douglas, T., & Devolder, K. (2013). Procreative altruism. *Journal of Medicine and Philosophy*,* 38*(4), 400–419.10.1093/jmp/jht022PMC390571023856478
 prospective parents have a moral reason to have a child whose existence can be expected to contribute more to the well‐being of others than any alternative child they could have. We recognize that the two principles can come into conflict, and that people who hold both principles will give them different weights. We also recognize that procreative choices involve other values, including the interests of parents and family. But we focus on procreative beneficence and altruism to illustrate the kinds of social dilemmas that widespread access to genetic modification and selection could create. We do not assume parents always know all of the relevant risks and benefits of procreative choices but we do assume that they have strong moral reasons to inform themselves of the science and moral trade‐offs their decisions involve.

### 4.1 Trade‐offs between mutually exclusive traits

As we argued above, some cognitive traits exist on a spectrum and some may be mutually exclusive. For example, some studies suggest that individuals who perform badly on tests that measure latent inhibition (an ability to block out irrelevant stimuli) do well on tests of creativity, and vice versa.46
Carson, S., & Peterson, J., & Higgins, D. (2003). Decreased latent inhibition is associated with increased creative achievement in high‐functioning individuals. *Journal of Personality and Social Psychology*,* 85*(3), 499–506.10.1037/0022-3514.85.3.49914498785
 In other words, these traits may be to some extent mutually exclusive—people are creative precisely because their mind wanders and they cannot block out seemingly irrelevant information.47
Gyngell, C., & Easteal, S. (2015). Cognitive diversity and moral enhancement. *Cambridge Quarterly of Healthcare Ethics*,* 24*(1), 66–74.10.1017/S096318011400031025473859



Assume it becomes possible to manipulate genes or select embryos in ways that influence these traits. Parents will face a tough choice. Being able to block out irrelevant details and concentrate is valuable in some circumstances and being creative is valuable in others. Whether either trait is good for someone is highly context dependent. This trade‐off makes it hard to apply procreative beneficence, as it is difficult for parents to pick the trait that will be overall best for their child. This trade‐off also makes it hard to apply procreative altruism. It seems likely that society benefits from having a mix of people who are creative and people who have good latent inhibition. Without knowing how other parents will act it is hard to know how to promote collective welfare when altering this trait.

Other traits may fit this pattern. For example, consider the trade‐off that exists between systemizing and empathy discussed above. Because each end of the spectrum is good in different circumstances and we do not know the exact circumstances facing our children, it is difficult to promote individual and collective welfare by altering them. When we generalize from a two‐person game to a choice faced by billions of people the optimal population mix becomes harder to figure out, and—even if it were discoverable—nearly impossible to achieve.

### 4.2 Trade‐offs between individually beneficial traits and collectively beneficial traits

Some psychological traits are clearly beneficial to individuals but will be collectively bad if possessed by all. Consider the choice to alter an embryo in a way that makes the child more extraverted. As indicated above, extraversion is heritable and people who score high on measures of extraversion tend to have better social relationships, are less prone to depression, and enjoy success in many different professions (most obviously, business). Extraverts are likely to be a bit happier, more effective in communicating to other members of a team, and better public speakers. They are also likely to make more money if their extraversion is combined with conscientiousness and high general intelligence.48
Gensowski, M. (2018). Personality, IQ, and lifetime earnings. *Labour Economics*,* 51*, 170–183.
 So it seems obvious that most parents motivated to improve the well‐being of their child would choose to make them extraverted.

Yet it's also plausible that having some introverts in a population is good for collective well‐being. Introverts are especially good at problem‐solving on their own—i.e., synthesizing and distilling information from collaborative work when they are alone.49
Cain, S. (2013). *Quiet: The power of introverts in a world that can't stop talking*. New York: Broadway.
 To illustrate why a combination of extraverts and introverts can be beneficial, consider the story of Apple Computers. Apple Computers was co‐founded by an introvert, Steve Wozniak, and an extravert, Steve Jobs. Wozniak was a more traditional computer geek—an introverted systematizer—while Jobs was a businessman with a creative vision—an extraverted showman. Both made crucial contributions to the company's success and to the development and adoption of personal computers. Their different cognitive styles worked together to the overall benefit of the company, the company's customers and the broader population.

The possibility of influencing traits along the introversion‐extraversion spectrum is therefore likely to lead to a dilemma between what is good for each (procreative beneficence) and what is good for all (procreative altruism). To the extent that we think both principles matter, regardless of whether we agree about how much they matter, this is a deep social dilemma. If everyone acts with the interest of their own children in mind it produces a result that is collectively suboptimal, assuming that procreative altruism has some non‐trivial weight. In other words, what is good for each may not be best (or even reasonably good) for all.

Similar dilemmas will arise for many other traits. Consider the choice to alter our children's genes to make them more compassionate than they would otherwise be. If everyone chose to engineer more compassionate (or affectively empathetic) children perhaps violence would decrease and altruism would increase. Other things equal, procreative altruism suggests this would be desirable. But from the standpoint of procreative beneficence there might be trade‐offs for children engineered in this way—for example, more compassionate people might lose some of the drive to achieve great things for themselves, like Olympic medals or artistic creations. However, suppose that a world in which everyone was highly compassionate would be best for all because of increased trust and reduced violence. Assuming this, procreative altruism implies we have reasons to select for highly compassionate children. However, if everyone were highly compassionate, this creates an incentive for defectors. People who are much more selfish than average may be able to thrive, benefiting from the trust and good will of others and giving little in return. In these circumstances procreative beneficence would instruct parents to produce children who are less compassionate. But if everyone acted like this it would be impartially worse for all. There is a coordination problem here. The best impartial result, from the standpoint of procreative altruism, can be produced only if everyone acts together—but this is beyond a parent's control.

In other words, in these simple and stylized examples there is no reason to expect the set of individual procreative choices to produce a social optimum. And there is no reason to assume that people will converge in their moral judgments about precisely what that optimum is, even if we can assume everyone will endorse procreative beneficence and procreative altruism. In sum, the ability to alter psychological traits will expose parents to a range of practical dilemmas involving trade‐offs. This raises the question of how we should regulate and protect cognitive diversity in an age of whole genome analysis, embryo selection, and gene editing.

## 5 THE NATURAL LOTTERY IS UNFAIR

In the face of this problem it might be tempting to ban genetic selection or enhancement. Even if such a ban were effective (which we have reason to doubt, given strong demand and the global reach of black markets), this would leave the balance of traits in the hands of the natural lottery. However, there is no reason to believe that nature has afforded human populations with optimum levels of diversity. The current distribution of traits has been shaped by natural selection. Natural selection works to promote the survival of individuals and genes. It does not aim to promote human well‐being or the collective good. It is clear that nature creates great inequality in traits that are relevant both at the level of personal and social welfare. There is no good reason to believe that normal human variation is optimal for the outcomes that matter.50
Op. cit note 42.
 Nor is there good reason to believe that leaving such choices to parents will be worse. At very least, parents are more likely than the natural lottery to promote their children's well‐being.

Furthermore, technological development is rapidly changing the environments to which humans are exposed. The way we interact with our environment today is very different from that just a few centuries ago. This has produced the problem of evolutionary mismatch, one version of which occurs when traits that were adaptive in ancestral conditions fail to promote either survival or well‐being in our current environment.51
Cofnas, N. (2016). A teleofunctional account of evolutionary mismatch. *Biology and Philosophy*,* 31*, 507–525.10.1007/s10539-016-9527-1PMC490110327358505
 For example, a person who scores high on neuroticism may very well have produced more surviving offspring, or contributed to the welfare of his tribe in an environment in which predators are a constant threat. Having an extreme fear of bugs or being disposed to worry about whether we've adequately prepared for the long winter ahead would be likely to be rewarded. But high levels of neuroticism today can result in clinical depression or anorexia.52
Lieberman, D. (2013). *The story of the human body*. New York: Vintage, Chapter 7.
 Natural selection is a slow process that works over several generations. Hence it is unlikely that it has equipped individuals or populations with the balance of personality traits that best promotes our flourishing today.

## 6 REGULATORY PARSIMONY

There is no way to avoid the questions raised in the previous sections. Failing to confront technologies that already enable us to select embryos, and will soon allow us to edit them is not a promising strategy, nor does it seem morally justifiable. Thinking about what kinds of people will exist and how we will create them is arguably our most important moral obligation.53
Savulescu, J., & Kahane, G. (2009). The moral obligation to produce children with the best chance of the best life. *Bioethics*,* 23*(5), 274–290; Gyngell, C., & Selgelid, M. (2016). Twenty‐first century eugenics. In L. Francis (Ed.), *The Oxford handbook of reproductive ethics* (pp. 141–158).Oxford: Oxford University Press; Anomaly, J. (2018). Defending eugenics: From cryptic choice to conscious selection. *Monash Bioethics Review*,* 35*(1–4), 24–35.10.1007/s40592-018-0081-2PMC609684929804244
 Some authors have argued that when the freedom to choose our children's characteristics creates a collective action problem, there is a prima facie case for government intervention.54
Gyngell, C., & Douglas, T. (2015). Stocking the genetic supermarket: Reproductive genetic technologies and collective action problems. *Bioethics*,* 29*(4), 241–250.10.1111/bioe.12098PMC440202924720568
 In a sense this is right: we cannot expect undirected private choice magically to produce an optimal distribution of goods in an economy or traits in a population. But we should always contrast whatever failures we expect from individual choice with the predictable failures of government intervention.55
Anomaly, J. (2015). Public goods and government action. *Politics, Philosophy, & Economics*,* 14*(2), 109–128.
 In fact, the dichotomy between undirected individual choice and coercive government rules is misleading. Social norms are important sources of social order and can deeply affect our individual preferences.

When we think about social interactions with many players and multiple equilibria, norms and laws can be thought of as rival ways of moving us from one equilibrium to another (socially superior) equilibrium. In small groups whose members have common values, low monitoring costs, and limited migration, collective action problems are typically best solved through informal norms. But in the kinds of collective action problems we're envisioning, millions of people in nation‐states and potentially billions of people around the world will be interacting in complex games that occur across time and space. In some cases, parents will move between different states or they will move between different communities within a state. The conditions for social norms to solve collective action problems will not always apply in these cases, unless people return to living in small and stable communities where norms can more powerfully sculpt behavior, including reproductive preferences.

This makes it hard to know what to do about far‐reaching collective action problems—for example, those that arise because many parents may prefer extraverts to introverts, or because all would prefer a population with more empathy, but each would prefer a child with less empathy than average. There is no algorithm that can tell us when we should rely on norms or laws to solve collective actions problems, though there are general rules of thumb that we can glean when we distinguish small number from large number cases, or cases with extensive externalities from those in which the consequences of our choices are mostly internalized.56
Anomaly, J., & Brennan, G. (2014). Social norms, the invisible hand, and the law. *University of Queensland Law Journal*,* 33*(2), 263–283.



We want to explore what Amy Gutmann calls the principle of regulatory parsimony, which she coined as a response to the kinds of collective action problems that arise with genetic engineering in particular, and synthetic biology more generally. Synthetic biology involves the use of computation and molecular biology to create and alter life.57
Venter, C. (2013). *Life at the speed of light: From the double helix to the dawn of digital life*. New York: Penguin.
 Some of the main uses include manipulating phage viruses capable of replacing antibiotics to fight bacterial infections, creating gene drives to eradicate vectors for infectious diseases, resurrecting extinct species, and altering the DNA of our children. According to Amy Gutmann, regulatory parsimony in the context of synthetic biology recommends

[O]nly as much oversight as is truly necessary to ensure justice, fairness, security, and safety while pursuing the public good. Regulatory parsimony is especially important in emerging technologies […] where the temptation to stifle innovation on the basis of uncertainty and fear of the unknown is particularly great. The blunt instruments of statutory and regulatory restraint may not only inhibit the distribution of new benefits, but can be counterproductive to security and safety by preventing researchers from developing effective safeguards.58
Gutmann, A. (2011). The ethics of synthetic biology: Guiding principles for emerging technologies. *Hastings Center Report*,* 41*(4), 17–22.10.1002/j.1552-146x.2011.tb00118.x21845917

This is a vague principle. One way to clarify regulatory parsimony is to think of it as implying that when legislation is necessary to prevent serious harms that arise from coordination problems, we should aim for simple rules that apply to all. In the cases we have in mind we should avoid micromanaging parental choices that shape the traits of their children. There are a number of reasons to endorse this principle that are not discussed by Gutmann. First, *complex* laws are often harder for ordinary people and small firms to navigate than they are for powerful people and large corporations. Similarly, wealthy people can typically afford to travel great distances or pay high costs to obtain goods that black markets are likely to provide if demand is strong, and goods that are made either illegal or expensive due to complex or otherwise burdensome laws. Complex laws, then, can harm the smallest companies and most vulnerable people.

Second, *too many* laws can crowd out social norms, which tend to be more sensitive to local conditions than government legislation.59
Ellickson, R. (1994). *Order without law*. Cambridge, MA: Harvard University Press.
 A small number of clear laws is less likely to stifle innovation. Innovation can occur both in the technology we seek to regulate but also in the norms and laws by which we might regulate the technology. We see regulatory parsimony as a tacit endorsement of federalism—the idea expressed by James Madison and others that, although we occasionally need a few simple rules at the level of a national government or an international assembly, we should also ensure that we allow local communities to experiment with different ways of achieving the desired results.

Third, laws can reduce liberty. The principle of regulatory parsimony in politics is analogous to the principle of the least restrictive alternative in ethics. Taking the least restrictive alternative is good because of the intrinsic value of liberty, but also because autonomy is promoted when people are confronted with alternatives and encouraged to develop the ability to make difficult decisions rather than relying on others to do so for them. Just as too many pharmaceutical regulations can result in a kind of learned helplessness by consumers,60
Flanigan, J. (2017). *Pharmaceutical freedom*. Oxford: Oxford University Press.
 an overly paternalistic approach to regulating genetic engineering can lead to false confidence in the wisdom of regulators and to a stunted capacity to make informed choices.

Finally, political processes produce incentives that result in a non‐random set of people who end up as regulators. These people will tend to have their own parochial views about which traits matter most, and what composition of future people would be best. Some regulators may possess wisdom that those who they regulate lack. But in many cases it will be the reverse and there are reasons to worry that giving regulators the power to micromanage procreative choices will leave some populations worse off than they would be with a greater degree of decentralized choice.

What would regulatory parsimony recommend for dilemmas like those discussed above? Choices that merely alter what some believe to be the optimal ratio of traits in the population should be permitted unless they produce a clear, uncompensated harm. Laws will be unnecessary if social norms evolve that reward people who have children with cognitive traits that are socially beneficial. Such norms could evolve because, as a population becomes skewed so that certain cognitive styles are both beneficial and scarce, parents might be more inclined to select children with these properties—in part because they will be rewarded with more successful lives due to the fact that their skills are in demand. This seems to be true for sex selection, at least in Western countries, where the ability to choose a child's sex doesn't seem to have resulted in any major imbalance and has been mainly chosen for purposes of family balancing.61
Savulescu, J., & Dahl, E. (2000). Sex selection and preimplantation diagnosis. *Human Reproduction*,* 15*(9), 1879–1880.10.1093/humrep/15.9.187910966977



More importantly, even if we can think of a hypothetical social optimum for a distribution of psychological traits and we agree on how to balance procreative beneficence and procreative altruism, it is not obvious that what experts come up with in estimating optimality will be right or that people will listen to them.

When in doubt, we should defer to the wishes of parents, as they typically have better incentives than regulators to figure out what traits will make their children flourish. As most parents would not maliciously produce children with traits that threaten the welfare of future people it may be superfluous to enact bans on selecting for anti‐social traits. But if parents refuse to take their responsibilities seriously by considering how their failure to select or alter an embryo might produce easily preventable harms to their child (or the future people whose welfare their child will affect), then it may be worth implementing regulations that limit these choices. We do not think free choices will always produce an optimal distribution of traits, and we are skeptical that a specific optimum exists, given reasonable diversity in the relative weight that different people place on widely shared values. But even if we could agree on a socially optimal distribution of traits we worry that states would be unlikely to achieve this optimum by micromanaging the procreative choices of parents. When legal regulations are appropriate to prevent serious harms we think they should be broad in scope and few in number.

## 7 CONCLUSION

Given the facts of scarcity and risk, we think there will be interesting trade‐offs when it comes to modifying genes associated with psychological traits. We have argued that as different traits can influence individual happiness and collective prosperity we should pay attention to the kinds of choices that new gene‐editing techniques will make possible. In the short and medium term it is likely that choosing a partner (or sperm and egg donor) wisely would be more effective and less risky than directly trying to edit genes to produce children with traits that are influenced by many genes interacting with each other. A lot of innovation in art and science depends on institutions, not (just) genes. But to the extent that genes influence who we are and what kinds of institutions we create there may be reasons to think about which social mechanisms we use for aggregating judgments, and which kinds of genetic enhancements might increase the welfare of each and the prosperity of all.

## CONFLICT OF INTEREST

The authors declares no conflict of interest.

